# A system of nonlinear set valued variational inclusions

**DOI:** 10.1186/2193-1801-3-318

**Published:** 2014-06-25

**Authors:** Yong-Kun Tang, Shih-sen Chang, Salahuddin Salahuddin

**Affiliations:** College of Statistics and Mathematics, Yunnan University of Finance and Economics, Kunming, Yunnan 650221 China; Department of Mathematics, Jazan University, Jazan, Kingdom of Saudi Arabia

**Keywords:** System of nonlinear set valued variational inclusions, Lipschitz continuity, Resolvent operators, Iterative sequences, Hilbert spaces

## Abstract

**Abstract:**

In this paper, we studied the existence theorems and techniques for finding the solutions of a system of nonlinear set valued variational inclusions in Hilbert spaces. To overcome the difficulties, due to the presence of a proper convex lower semicontinuous function *ϕ* and a mapping *g* which appeared in the considered problems, we have used the resolvent operator technique to suggest an iterative algorithm to compute approximate solutions of the system of nonlinear set valued variational inclusions. The convergence of the iterative sequences generated by algorithm is also proved.

**AMS Mathematics subject classification:**

49J40; 47H06

## Introduction

It is well known that variational inequality theory and complementarity problems are very powerful tools of current mathematical technology. In recent years, the classical variational inequality and complementarity problems have been extended and generalized to study a large variety of problems arising in economics, control problems, contact problems, mechanics, transportation, equilibrium problems, optimization theory, nonlinear programming, transportation equilibrium and engineering sciences, see (Aubin 1982; Baiocchi and Capelo 1984; Chang 1984; Giannessi and Maugeri 1995). Hassouni and Moudafi 2001 introduced and studied a class of mixed type variational inequalities with single valued mappings which was called variational inclusions. Since many authors have obtained important extension generalizations of the results in (Hassouni and Moudafi 2001) from various directions, see (Agarwal et al. 2011; Fang et al. 2005; Kassay and Kolumban 2000; Petrot 2010). Verma 1999; 2001a introduced and studied some system of variational inequalities with iterative algorithms to compute approximate solutions in Hilbert spaces.

Inspired and motivated by the research work going on this field, in this works, the methods for finding the common solutions of a system of nonlinear set valued variational inclusions involving different nonlinear operators and fixed point problem are considered and studied, via proximal method in the framework of Hilbert spaces.

Since the problems of a system of a nonlinear set valued variational inequalities and fixed point are both important, the results present in this paper are useful and can be viewed as an improvement and extension of the previously known results appearing in literature, which are improves the results of Chang et al. 2007 and also extends the results of Verma 2001b; 2002, Ahmad and Salahuddin 2012, Ding and Luo 2000, Inchan and Petrot 2011, Kim and Kim 2004, Kim and Hu 2008, Nie et al. 2003 and Suantai and Petrot 2011, etc.

Let *H* be a real Hilbert space whose inner product and norm are denoted by 〈·,·〉 and ∥·∥ respectively and *K* be a nonempty closed convex subset of *H*. Let *CB*(*H*) be the family of all nonempty closed convex and bounded sets in *H* and *ϕ*:*H*→(−*∞*,+*∞*) be a proper convex lower semicontinuous function on *H*. Let *N*_*i*_:*H*×*H*→*H* be a nonlinear function, *g*_*i*_:*K*→*H* be a nonlinear operator, *A*_*i*_,*B*_*i*_:*K*→*CB*(*H*) be the nonlinear set valued mappings and let *r*_*i*_ be a fixed positive real number for each *i*=1,2,3. Set *The system of nonlinear setvalued variational inclusions involving three different nonlinear operators* is defined as follows:

Find  such that
1

We denote the set of all solutions  of problem (1) by SNSVVID.

We first recall some basic concepts and well known results.

### Definition 1

A mapping *g*:*H*→*H* is said to be

(i) *monotone*, if
(ii) *strictly monotone, if g is monotone and*
(iii) *υ-strongly monotone, if there exists a constant υ>0 such that*
(iv) *Lipschitz continuous, if there exists a constant υ>0 such that*

### Definition 2

A set valued mapping *A*:*H*→2^*H*^ is said to be *υ-strongly monotone*, if there exists a constant *υ*>0 such that


### Definition 3

A set valued mapping *A*:*H*→*CB*(*H*) is said to be *τ*-Lipschitz continuous if there exists a constant *τ*>0 such that


where (·,·) is the Hausdorff metric on CB().

### Definition 4

(Brezis 1973)

If *M* is maximal monotone operator on *H* then for any *λ*>0*the resolvent operator associated with M is defined by*

It is well know that a monotone operator is maximal iff its resolvent operator is defined every where. Furthermore the resolvent operator is single valued and nonexpansive. In particular the subdifferential *∂ϕ* of a proper convex lower semicontinuous function *ϕ*:*H*→(−*∞*,+*∞*) is a maximal monotone operator.

### Lemma 1

(Brezis 1973) The points *u*,*z*∈*H* satisfies the inequality


if and only if


where  is a resolvent operator and *λ*>0 is a constant.

For any  is nonexpansive, i.e.,


Assume that *g*:*H*→*H* is a surjective mapping and from Lemma 1 and (1) we have the following proximal point problem:
2

provided *K*⊂*g*_*i*_(*H*) for each *i*=1,2,3.

### Lemma 2

(Weng 1991)

Let {*a*_*n*_},{*b*_*n*_} and {*c*_*n*_} be three sequences of nonnegative real numbers such that


where *n*_0_ is a nonnegative integer, {*t*_*n*_} is a sequence in (0,1) with  and . Than *a*_*n*_→0 as *n*→+*∞*.

### Definition 5

Let *A*,*B*:*H*→2^*H*^ be set valued mappings and *N*:*H*×*H*→*H* be a nonlinear mapping.

(i) *N* is said to be *A-strongly monotone with respect to the first argument*, if there exists a constant *υ*>0 such that for all *x*,*y*∈*H*
(ii) *N is said to be B-relaxed monotone with respect to the second argument, if there exists a constantξ*>0 such that for all *x*,*y*∈*H*,*v*_1_∈*B*(*x*),*v*_2_∈*B*(*y*)

## Main results

We begin with some observations which are related to the problem (1).

### Remark 1

If (*x*^∗^,*y*^∗^,*z*^∗^)∈ SNSVVID, by (2) we have that
3

provided *K*⊂*g*_1_(*H*).

Consequently if *S* is a Lipschitz mapping such that *x*^∗^∈*F*(*S*), then it follows from (3) that
4

By virtue of (4) and Nadler’s Theorem (Nadler 1969), we suggest the following iterative algorithm.

**Algorithm 1** Let *ε*_*n*_ be a sequence of nonnegative real number with *ε*_*n*_→0 as *n*→*∞*. Let *r*_1_,*r*_2_,*r*_3_ be three given positive real numbers in (0,1). For arbitrary chosen initial *x*_0_∈*H*, compute the sequences {*x*_*n*_},{*y*_*n*_} and {*z*_*n*_} in *H*, such that
5

where
6

and {*α*_*n*_} is a sequence in (0,1) and *S*:*H*→*H* is a mapping.

### Theorem 1

Let *K* be a nonempty closed and convex subset of a real Hilbert space *H* and *ϕ*:*H*→(−*∞*,+*∞*) be a proper convex lower semicontinuous function. Let *A*_*i*_:*H*→2^*H*^ be a *μ*_*i*_-Lipschitz continuous mapping with *μ*_*i*_<1 and *B*_*i*_:*H*→2^*H*^ be a *σ*_*i*_-Lipschitz continuous mapping with *σ*_*i*_<1, *i*=1,2,3. Let *N*_*i*_:*H*×*H*→*H* be a *ρ*_*i*_-Lipschitz continuous with respect to the first variable and *η*_*i*_-Lipschitz continuous with respect to the second variable and *N*_*i*_ be *A*_*i*_-strongly monotone with constant *υ*_*i*_>0 and *B*_*i*_-relaxed monotone with constant *ξ*_*i*_>0, *i*=1,2,3. Let *g*_*i*_:*H*→*H* be a *λ*_*i*_-strongly monotone and *γ*_*i*_-Lipschitz continuous mapping, *i*=1,2,3. Let *S*:*H*→*H* be a *τ*-Lipschitz continuous mapping with 0<*τ*≤1. If , and the following conditions are satisfied:

(i)

where ;

(ii)
(iii) for each *i*=1,2,3

where
7

where *M*= sup*n*≥1*ε*_*n*_.

(iv) {*α*_*n*_}⊂(0,1) such that .

Then the sequences {*x*_*n*_},{*y*_*n*_},{*z*_*n*_},{*u*_*n*,*i*_},{*v*_*n*,*i*_} suggested by Algorithm 1 converge strongly to  respectively, and  SNSVVID, *x*^∗^∈*F*(*S*).

**Proof.** Let  and *x*^∗^∈*F*(*S*). By (2) and (4) we have
8

Consequently, by (5) and (6), we have
9

Since *N*_1_(·,·) is *ρ*_1_-Lipschitz continuous with respect to the first variable and *η*_1_-Lipschitz continuous with respect to the second variable, and *A*_1_ is *μ*_1_-Lipschitz continuous, and *B*_1_ is *σ*_1_-Lipschitz continuous, we have
10

Since *N*_1_ is *A*_1_-strongly monotone with constant *υ*_1_>0 and *B*_1_-relaxed monotone with constant *ξ*_*i*_>0, it follows from (10) that


i.e.,
11

where


Note that
12

Since *g*_2_ is *λ*_2_-strongly monotone and *γ*_2_-Lipschitz continuous mapping, we have
13

where .

On the other hand, by (2) and (5), we have
14

In view of the assumptions of *N*_2_,*A*_2_,*B*_2_, *g*_2_ and by using the same method as given in the proofs in (11) and (13), we can obtain that
15

where


and
16

From (15), (16) and (14), we have
17

Combining (12), (13) and (17) we obtained
18

Observe that
19

and in view of (2) and (5), we have
20

By using the assumptions on *N*_3_,*A*_3_,*B*_3_ and *g*_3_, we have
21

where
2223

Substituting (21) and (22) into (20), we have
24

Combining (19), (23) and (24), it yields that
25

This imply that
26

Substituting (26) into (18) we have
27

that is
28

From (11) and (28), we get
29

On the other hand, since *g*_1_ is *λ*_1_-strongly monotone and *γ*_1_-Lipschitz continuous mapping, we have


i.e.,
30

Similarly, we have
31

Substituting (28) into (31), we have
32

Set
33

Substituting (30), (31), (32) and (33) into (9), we get
34

Since


letting , then we have *ℓ*_*n*_≤*ℓ*. Therefore from (34) we have that
35

By condition (iii)
36

this imply that
37

that is
38

Put
39

By the assumption that 0<*τ*≤1, it follows that


This imply that *t*_*n*_∈(0,1). From assumption (iv) we have


These show that all conditions in Lemma 2 are satisfied. Hence *x*_*n*_→*x*^∗^ as *n*→*∞*. Consequently from (26) and (28), we have *z*_*n*_→*z*^∗^ and *y*_*n*_→*y*^∗^ as *n*→*∞*, respectively. Moreover since *A*_*i*_ is *μ*_*i*_-Lipschitz continuous and *B*_*i*_ is *σ*_*i*_-Lipschitz continuous with *μ*_*i*_<1, *σ*_*i*_<1, we can also prove that {*u*_*n*,*i*_} and {*v*_*n*,*i*_}, *i*=1,2,3 are Cauchy sequences. Thus there exists  such that  as *n*→*∞*. Moreover by using the continuity of mappings , *i*=1,2,3, it follows from (5) that


Hence from Lemma 2 it follows that  SNSVVID Finally we prove that  and  Indeed we have


That is . Since *A*_1_(*y*^∗^)∈*CB*(*H*), we must have . Similarly we can show that  and  This complete the proof. â–
